# Predicting antimicrobial mechanism-of-action from transcriptomes: A generalizable explainable artificial intelligence approach

**DOI:** 10.1371/journal.pcbi.1008857

**Published:** 2021-03-29

**Authors:** Josh L. Espinoza, Chris L. Dupont, Aubrie O’Rourke, Sinem Beyhan, Pavel Morales, Amy Spoering, Kirsten J. Meyer, Agnes P. Chan, Yongwook Choi, William C. Nierman, Kim Lewis, Karen E. Nelson

**Affiliations:** 1 J. Craig Venter Institute, La Jolla, CA, United States of America; 2 Department of Applied Sciences, Durban University of Technology, Durban, South Africa; 3 NovoBiotic Pharmaceuticals, Cambridge, MA, United States of America; 4 Department of Biology, Northeastern University, Boston, MA, United States of America; 5 J. Craig Venter Institute, Rockville, MD, United States of America; Icahn School of Medicine at Mount Sinai, UNITED STATES

## Abstract

To better combat the expansion of antibiotic resistance in pathogens, new compounds, particularly those with novel mechanisms-of-action [MOA], represent a major research priority in biomedical science. However, rediscovery of known antibiotics demonstrates a need for approaches that accurately identify potential novelty with higher throughput and reduced labor. Here we describe an explainable artificial intelligence classification methodology that emphasizes prediction performance and human interpretability by using a Hierarchical Ensemble of Classifiers model optimized with a novel feature selection algorithm called *Clairvoyance*; collectively referred to as a CoHEC model. We evaluated our methods using whole transcriptome responses from *Escherichia coli* challenged with 41 known antibiotics and 9 crude extracts while depositing 122 transcriptomes unique to this study. Our CoHEC model can properly predict the primary MOA of previously unobserved compounds in both purified forms and crude extracts at an accuracy above 99%, while also correctly identifying darobactin, a newly discovered antibiotic, as having a novel MOA. In addition, we deploy our methods on a recent *E*. *coli* transcriptomics dataset from a different strain and a *Mycobacterium smegmatis* metabolomics timeseries dataset showcasing exceptionally high performance; improving upon the performance metrics of the original publications. We not only provide insight into the biological interpretation of our model but also that the concept of MOA is a non-discrete heuristic with diverse effects for different compounds within the same MOA, suggesting substantial antibiotic diversity awaiting discovery within existing MOA.

## Author summary

As antimicrobial resistance is on the rise, the need for compounds with novel targets or mechanisms-of-action [MOA] are of the utmost importance from the standpoint of public health. A major bottleneck in drug discovery is the ability to rapidly screen candidate compounds for precise MOA activity as current approaches are expensive, time consuming, and are difficult to implement in high-throughput. To alleviate this bottleneck in drug discovery, we developed a human interpretable artificial intelligence classification framework that can be used to build highly accurate and flexible predictive models. In this study, we investigated antimicrobial MOA through the transcriptional responses of *Escherichia coli* challenged with 41 known antibiotic compounds, 9 crude extracts, and a recently discovered (circa 2019) compound, darobactin, with novel MOA activity. We implemented a highly stringent Leave Compound Out Cross-Validation procedure to stress-test our predictive models by simulating the scenario of observing novel compounds. Furthermore, we developed a versatile feature selection algorithm, *Clairvoyance*, that we apply to our hierarchical ensemble of classifiers framework to build high performance explainable machine-learning models. Although the methods in this study were developed and stress-tested to predict the primary MOA from transcriptomic responses in *E*. *coli*, we designed these methods for general application to any classification problem and open-sourced the implementations in our *Soothsayer* Python package. We further demonstrate the versatility of these methods by deploying them on recent *Mycobacterium smegmatis* metabolomic and *E*. *coli* transcriptomics datasets to predict MOA with high accuracy.

## Introduction

As antibiotic resistant pathogens have increasingly emerged [1,2], the discovery of new antimicrobials has lagged [3,4] despite previous efforts in screening hundreds of thousands of compounds [5]. Many of the screened compounds are either identical to known drugs, close analogs thereof, or have the same molecular targets [6]. Despite the wide variety of utilized antibiotics, many of these appear to collapse into 6 distinct mechanisms-of-action [MOA] based on pure enzyme inhibition assays. Progress within antibiotic discovery has been relatively slow [7] and the discovery of new antibiotics within existing MOA [8] constitutes a vanishingly low percentage of screened compounds [3,4]. This redundant discovery and diminishing returns of new chemical entities underpins declining industry efforts in screening for new antimicrobial drugs and a desire for disruptive new approaches.

One of the barriers to finding new chemical entities with novel biological targets is the problem of dereplication; the determination of a compound’s primary MOA is time-consuming and often results in rediscovering a previously observed compound from a known MOA. The typical screening for antibiotics entails bacterial growth inhibition assays followed by macromolecular synthesis assay [9], with the former defining antibacterial activity and the latter determining the primary MOA [7]. Growth inhibition assays are easily automated and performed in high throughput [10]. An automated method to screen new antimicrobial compounds in high-throughput for both predicted MOA and similarity to known antibiotics as an intermediary step would obviate a major bottleneck in the path from drug discovery to clinical trials. Efforts to utilize more detailed whole cell bioreporter methods include large scale mutant library screening [11], whole cell imaging [12–14], proteome profiling [15,16], transcriptomics [12,17–20], and metabolomics [21]. Relative to the other approaches, transcriptome profiling benefits from capturing broad gene expression information relative to input labor. In previous MOA predictive modeling studies, accuracy estimated was occasionally absent [20], difficult to reproduce [19], or lacked robustness on held-out compounds [19]. More recent studies did validate their models but did not evaluate their models on unobserved compounds [12,20,21]. The desired method should have high prediction accuracy validated on compounds not included in the training data and, therefore, unobserved by the model. An approach that abides by this stringency is Leave Compound Out Cross-Validation [LCOCV] where all instances of a compound are reserved for a testing set and the remaining compounds are used for model training; thus, demonstrating predictive performance on unobserved compounds. Even though a large set of transcriptomic data has been accrued in this field, the datasets have not been utilized effectively to build predictive MOA classification models, presenting a unique opportunity for exploiting recent advancements in artificial intelligence [AI] and machine learning.

As AI broadly mimics the cognitive abilities of the human mind, machine learning, a subset of AI, focuses on the ability of machines to receive input and adapt to information for a variety of tasks including predictive modeling and data mining for diagnostic genes. Machine-learning algorithms require large amounts of high-quality training data from intelligently designed experiments to effectively learn latent patterns that describe phenomena; in the case of this study, patterns within differential gene expression [DGE] profiles that can discriminate MOA. However, many high-performance models such as deep neural networks are difficult to interpret in a biological context where transparency in diagnostic decisions are paramount for reliable clinical applications. Explainable AI, often abbreviated XAI, is an effort to produce human interpretable models while maintaining a high level of learning performance [22]. Interpretability in the context of AI translates to a detailed understanding of a model’s decision-making process. Although XAI cannot directly explain hitherto unknown biological phenomena, it can be used synergistically to guide research endeavors with domain expertise which, in turn, produce more realistic models resulting in a positive feedback-loop of information gain.

Given the proper training data, XAI can be leveraged to address two major questions of biotechnological and fundamental importance in antibiotic discovery. First, can XAI utilize whole transcriptome responses to predict the primary MOA of a compound or culture extract with high accuracy? In this scenario, antibacterial compounds or extracts that defy classification potentially represent new chemical entities with novel molecular targets or MOA; a major goal of biomedical science. Of seemingly lesser but potentially greater impact, does an examination of these responses within compounds of the same MOA reveal established MOA categories to be discrete entities or rather a spectrum of biological responses? In the latter case, compounds categorized within existing MOA but with unique transcriptional responses may represent new chemical entities that would have been discarded erroneously using traditional approaches.

## Results

### Antimicrobial mechanism of action training data compounds and producer-strain extracts

Our training dataset consists of 41 antibiotic majority FDA-approved compounds representing 6 MOA including inhibitors for protein-, DNA-, RNA-, cell-wall-, cell-membrane-, and fatty-acid-synthesis (Tables 1 and S1), which were chosen to maximize coverage of MOA and chemoinformatic space. The challenge experiments were conducted in *Escherichia coli* strain *W0153*, which has a permeable outer-membrane susceptible to large hydrophobic antibiotics [23], allowing us to investigate the effects of more antibiotic compounds at lower concentrations than wild-type strains, therefore, reducing the likelihood of off-target effects that could trigger secondary MOA activities. For each compound, at least triplicate challenges were conducted and transcriptomes were sequenced to analyze gene expression profiles.

**Table 1:** Training data for pure compounds and producer-strain extracts relative to MOA. Number of compounds, samples, and pairwise DGE profiles for pure compounds and producer-strain extracts relative to individual MOA.

	Pure Compounds			Producer-strain Extracts		
	Compounds	Samples	Pairwise DGE Profiles	Compounds	Samples	Pairwise DGE Profiles
**MOA**						
**cell-membrane**	2	11	33	0	0	0
**cell-wall**	12	61	178	4	18	54
**dna-synthesis**	10	52	171	2	7	21
**fatty-acid-synthesis**	3	12	36	0	0	0
**protein-synthesis**	9	42	126	2	6	18
**rna-polymerase**	4	20	58	2	6	18

Historically, the majority of antibiotics have been discovered and isolated by fermenting soil bacteria. Hence for nine compounds, we also included crude extracts from organisms producing a specific antibiotic compound (called “producer-strain extracts” herein) to prepare our models for high-throughput discovery pipelines of microbial extracts, which would obviate the time-consuming chemical purification.

The specific machine learning problem addressed in this study is to robustly predict the MOA of a compound unobserved by the model using gene expression data generated from microbes treated with said compound. An added constraint of this overarching task is to ensure maximum model interpretability without sacrificing model performance and these objectives are evaluated by simulating predictive performance on novel compounds *in silico* (i.e., LCOCV). As machine learning algorithms benefit greatly from more high-quality training data, we used pairwise DGE profiles (instead of summary statistics) to maximize the number of observations while simultaneously accounting for bias between sampling and providing prediction error profiles. This simple procedure increased our available training data from 235 observations to 713 observations and, thus, providing more information that can be used for modeling (Table 1). With these 713 pairwise DGE profiles, we used 3065 protein-coding genes as features to increase opportunities for downstream interpretability and potential *post hoc* validation experiments.

### Feature selection to optimize held-out compound classification performance

Machine learning models tend to overfit when the number of features vastly exceeded the number of observations; in this case, genes and biological samples, respectively. The training data dimensionality is not ideal for even simple binary classification models, let alone 6 imbalanced classes, thus, it was not surprising to find that most traditional classification models performed poorly (<90% LCOCV accuracy) (Table 2). Our solution to overcome this dimensionality obstacle was to develop the *Clairvoyance* feature selection algorithm as a means for curating gene sets that could robustly discriminate the primary MOA of DGE profiles. The objective function implemented in *Clairvoyance* maximizes the accuracy of custom (or stochastic) cross-validation pairs by iteratively enriching the subset of predictive features (e.g., genes). This iterative enrichment denoises the dataset with respect to a specific classification task resulting in a smaller feature set with reduced potential for model overfitting (see *S1 Methods*). In the case of this study, the *Clairvoyance* algorithm iteratively refines the gene sets to maximize the MOA classification accuracy of unobserved compounds provided as the test set in our custom LCOCV pairs to simulate the performance on novel compounds.

**Table 2:** Model performance using several supervised machine-learning algorithms. Various machine-learning algorithms were evaluated using the entire feature set (n = 3065 genes) and the *Clairvoyance*-optimized feature set (*GeneSet*_*y1-y5*_, n = 399 genes) with the same LCOCV pairs. Performance metrics for each LCOCV set include accuracy, precision, recall, and F1 score. LCOCV refers to Leave Compound Out Cross Validation where we remove all instances of a compound from the data used to fit the model (training data) and evaluate performance on the held-out compound profiles (testing data) (see *Materials and Methods*).

	Clairvoyance feature selection [N = 399 Genes]				No feature selection [N = 3065 Genes]			
	Accuracy	F1 Score	Precision	Recall	Accuracy	F1 Score	Precision	Recall
**Classifier**								
**CoHEC**	0.999	0.983	0.983	0.982	0.749	0.693	0.715	0.682
**Logistic Regression**	0.880	0.829	0.856	0.817	0.793	0.732	0.763	0.723
**Random Forest**	0.792	0.719	0.768	0.708	0.742	0.659	0.703	0.645
**K-Nearest Neighbors**	0.714	0.568	0.617	0.546	0.636	0.506	0.561	0.481
**Support Vector Machine**	0.798	0.722	0.778	0.704	0.694	0.616	0.668	0.600
**Naive Bayes (Gaussian)**	0.698	0.582	0.623	0.561	0.429	0.302	0.389	0.274
**AdaBoost**	0.333	0.308	0.333	0.301	0.339	0.277	0.333	0.261
**Neural Network**	0.872	0.785	0.815	0.773	0.741	0.635	0.683	0.619

We leveraged *Clairvoyance* feature selection with a multiclass version of a logistic regression model predicting MOA using a one-vs-rest architecture. Without feature selection, this model predicts the MOA from unobserved compounds with a LCOCV accuracy of 79.3% (Table 2). With feature selection designed for multiclass predictions, *Clairvoyance* was able to identify 98 genes (*GeneSet*_*Multiclass*_) that could predict MOA from unobserved compounds with a LCOCV accuracy above 95% (Table 3). Although the performance of this model is high, we wanted to extend our methods to a hierarchical framework to better understand the decision-making process and maximize the amount of available information.

**Table 3:** Evaluating external datasets using CoHEC models. MOA prediction accuracy and performance when applying our methods to the data from Zoffmann et al. 2019 and Zampieri et al. 2018 and the methods from Hutter et al. 2004 on our dataset. In all cases, LCOCV was used for evaluating model performance for each individual observation (e.g. pairwise DGE profile), each cross-validation set (e.g. held out teixobactin), and using various majority voting schemes (see *Materials and Methods*). CPD is an abbreviation for compound. *Indicates protein-synthesis sub-MOA classification (30S/50S).

Dataset	Model	Organism	Feature Set Label	Feature Type	Features	MOA	CPD	Individual Pairwise Profiles Accuracy	LCOCV Test Set Accuracy	Majority Voting (Hard) Accuracy	Majority Voting (Soft) Accuracy	Data Source
This study (All MOA)	CoHEC	Escherichia coli (W01573)	GeneSet_y1-y5	Gene	399	6	41	0.9972	0.9986	1	1	https://www.ncbi.nlm.nih.gov/bioproject/?term=PRJNA532938
This study (All MOA)	Clairvoyance-optimized multiclass logistic regression	Escherichia coli (W01573)	GeneSet_y1-y5	Gene	399	6	41	0.85714286	0.88017911	0.86440678	0.89830508	https://www.ncbi.nlm.nih.gov/bioproject/?term=PRJNA532938
This study (30S/50S)	Clairvoyance-optimized binary logistic regression	Escherichia coli (W01573)	GeneSet_30S/50S	Gene	7	2*	9	0.9691358	0.96153846	1	1	https://www.ncbi.nlm.nih.gov/bioproject/?term=PRJNA532938
This study (All MOA)	Clairvoyance-optimized multiclass logistic regression	Escherichia coli (W01573)	GeneSet_Multiclass	Gene	98	6	41	0.95936	0.967735	0.983051	1	https://www.ncbi.nlm.nih.gov/bioproject/?term=PRJNA532938
This study (All MOA)	Support vector machine (Hutter et al. 2004 Methods)	Escherichia coli (W01573)	-	Gene	-	6	41	0.758	-	-	-	-
Zoffmann et al. 2019	CoHEC	Escherichia coli (BW25113)	GeneSet_Zoffmann	Gene	35	4	16	1	1	1	1	https://www.ncbi.nlm.nih.gov/geo/query/acc.cgi?acc=GSE110137
Zampieri et al. 2018 (*reference_t0*)	CoHEC	*Mycobacterium smegmatis*	MetaboliteSet_Zampieri-t0	Metabolite	492	18	62	0.949	0.949	0.977	0.991	https://www.ebi.ac.uk/biostudies/studies/S-BSST113
Zampieri et al. 2018 (*reference_solvent*)	CoHEC	*Mycobacterium smegmatis*	MetaboliteSet_Zampieri-solvent	Metabolite	494	18	62	0.882	0.882	0.954	0.963	https://www.ebi.ac.uk/biostudies/studies/S-BSST113

### Hierarchical framework for multiclass classifications

With inspiration from the mechanisms of human cognition and the applications to automated facial recognition [24], we sought to decompose the complex task of multiclassification into a multilayered path of simple binary tasks [25]. We have developed a flexible framework for implementing Hierarchical Ensemble of Classifiers [HEC] models and their *Clairvoyance*-optimized counterpart [CoHEC]. Our basic HEC model approach implements a hierarchical ensemble of binary classifiers through a single graphical model with 3 degrees of flexibility for each sub-model decision node: (1) a custom feature set optimized for a simple binary classification task; (2) a unique classification algorithm with hyperparameters that most effectively discriminates the sub-model-specific decision paths; and (3) the relationship between sub-models can be data-driven or assigned *a priori*.

**Fig 1:**
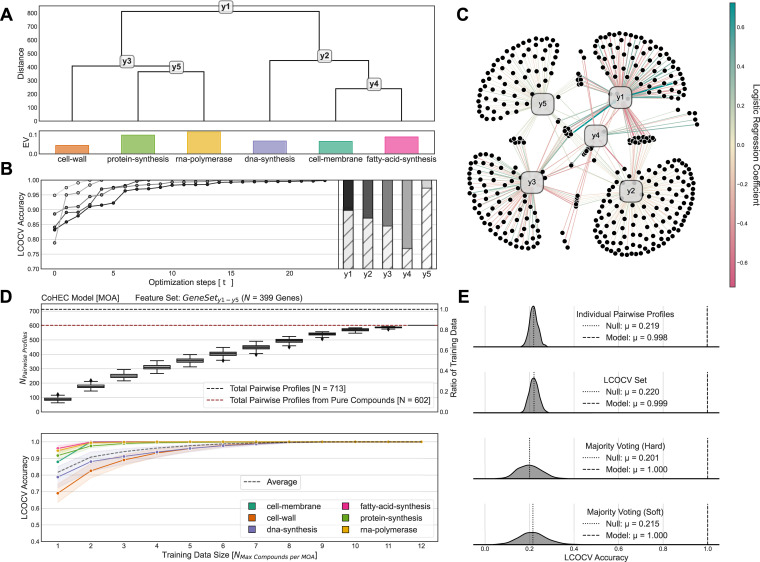
MOA classification performance and model benchmarking. A) The empirically determined structure of the CoHEC model calibrated to predict the MOA of an unobserved antibacterial compound based on the transcriptional change profiles of *E*. *coli*. The colored bar chart below the dendrogram shows the explained variance of the eigenprofile for each MOA. B) The influence of the *Clairvoyance* optimization algorithm for feature selection on model performance at each of the 5 sub-model decision points. Optimization step (*t* = 0) corresponds to using all available gene features, while each optimization step removes low information features during each consecutive iteration. The column chart shows the original baseline accuracy (lower) with all 3065 gene features and the effects of *Clairvoyance* optimized feature selection (upper). C) Network visualization of genes feature sets, determined by *Clairvoyance*, used by each sub-model decision point of the CoHEC model. The edge width represents the coefficient magnitude in each fitted Logistic Regression sub-model with the sign reflected by the color (positive = teal, negative = rose). D) Benchmarking of CoHEC model performance (N = 500 permutations without repetition) showing (upper) the number of compounds included during (lower) LCOCV evaluation relative to performance. Error bars represent standard error of mean. E) Kernel density of LCOCV accuracy for CoHEC null model (N = 500 permutations without repetition) and dashed horizontal lines representing actual CoHEC model performance.

The graphical structure of our CoHEC model (Figs 1A and S2) is entirely data-driven to demonstrate the autonomous abilities of our XAI methodology by solely using emergent patterns within the training dataset in relation to the labeled classes. In other words, we do not predefine the graphical structure or gene sets using curated databases or domain knowledge (although, this functionality is supported) and instead allow the data to guide such parameter choices. Optimization of the gene feature set for each sub-model using *Clairvoyance* (*GeneSet*_*yk*_ where *k* ranges from sub-models 1–5) boosted LCOCV accuracy substantially; between 10–23% in most cases and all cases resulting in left-out compound accuracies greater than 99% (Fig 1B and S2 Table). Several estimators were evaluated, optimized, and tuned for each sub-classification task but logistic regression models were the exemplar in all cases. While a few genes are shared between various pairs of sub-models, none of the 399 unique genes from *GeneSet*_*y1-y5*_ used in the CoHEC model were universal to all sub-models reinforcing the notion that each sub-model is task specific (Fig 1C and S2 and S3 Tables). Of these 399 genes in our CoHEC model, there were 87 of the 98 genes (88.8%) in *GeneSet*_*Multiclass*_ overlapping (S6 Fig) and, thus, demonstrating the ability of *Clairvoyance* to identify emergent patterns within the data despite different model architectures. Interestingly, none of the MOA enzymatic targets were selected by *Clairvoyance* as discriminative features further endorsing our data-driven approach because the discriminating patterns were unknown *a priori*.

Our hierarchical framework provides a seamless avenue for introducing additional classification layers *post hoc* to a fitted model. For example, our CoHEC model was initially designed to classify 6 MOA categories but we’ve augmented the model with an additional layer to predict sub-MOA for 30S/50S subunit protein-synthesis inhibitors to showcase this functionality. In our CoHEC model, protein-synthesis is discriminated from rna-polymerase inhibitors by sub-model *y5* using a subset of 92 genes (*GeneSet*_*y5*_). To demonstrate how the information content in our hierarchical model is nested, we used *Clairvoyance* with the 92 genes from sub-model *y5* to identify an additional feature set that could robustly discriminate 30S from 50S protein-synthesis inhibitors (S1 Table). This approach predicted the target subunit of protein-synthesis inhibitors with a LCOCV accuracy greater than 96% using a subset of only 7 genes (*GeneSet*_*30S/50S*_) (S5E and S5F Fig and S2 and S3 Tables) from sub-model *y5* and only overlapped with gene sets from sub-models in protein-synthesis decision path.

### Model evaluation and benchmarking

The structure of our training data and our representation of differential expression allowed us to evaluate unobserved compound accuracy on 3 hierarchical abstractions. For our CoHEC model using *GeneSet*_*y1-y5*_, we have the following evaluation: (1) the accuracy of individual pairwise DGE profiles using LCOCV (99.72%), (2) the mean accuracy for each LCOCV test set (99.86%), and (3) the majority voting consensus prediction for a compound from multiple individual predictions (100%) as shown in Tables 2,3,S4, and S7, and described in detail with *Materials and Methods*. Consensus predictions from our CoHEC model using individual predictions grouped by LCOCV test sets can be accomplished via soft majority voting with sub-model probabilities or hard majority voting with only terminal predictions; majority voting is a method that combines the results of multiple predictions into a single prediction. Regardless of the voting scheme, the CoHEC model achieves 100% accuracy for predicting the primary MOA from unobserved compounds despite the draconian method of leaving out all instances of a compound when fitting the model during LCOCV (S3 Fig and Tables 3 and S7). This methodology out-performs that of previous studies [14,19,21] despite our usage of a far more stringent accuracy validation method.

We compared our CoHEC model performance to similar models and methods to assess performance gains. As mentioned prior, we used *Clairvoyance* with a multiclass logistic regression and obtained accuracies greater than 96% using *GeneSet*_*Multiclass*_ (Tables 2 and S7). To test whether our CoHEC model can outperform a standard multiclass model given the same input data, we evaluated another multiclass logistic regression but instead of using *GeneSet*_*Multiclass*_ we used the 399 genes from *GeneSet*_*y1-y5*_ which was designed for a hierarchical architecture. The multiclass version of our CoHEC model performed with a LCOCV accuracy between 85.7% and 89.8% depending on evaluation method (Tables 3 and S7). Although hierarchical feature selection that is designed for a multiclass model (*GeneSet*_*Multiclass*_) or adapted from a CoHEC model (*GeneSet*_*y1-y5*_) improves the classification performance when compared to a standard multiclass model without feature selection (LCOCV accuracy = 79.3%, Table 2), these methods cannot compete with the synergy of feature selection and hierarchical classifications implemented in our CoHEC model.

In addition to evaluating (Tables 3 and S7) and benchmarking (Fig 1D and 1E) our CoHEC model’s ability to predict MOA, we also tested the performance of the following models: (1) multiclass logistic regression model using *GeneSet*_*y1-y5*_ predicting MOA (S5A and S5B Fig); (2) *Clairvoyance-*optimized multiclass logistic regression model (*GeneSet*_*Multiclass*_) predicting MOA (S5C and S5D Fig); and (3) the *Clairvoyance*-optimized binary logistic regression model predicting 30S/50S protein-synthesis inhibitors (*GeneSet*_*30S/50S*_) (S5E and S5F Fig). The null LCOCV accuracy of our MOA predictive models had a similar range 20% - 24% (Figs 1E, S5B and S5D) which is only slightly above the expected null accuracy of 16.6% given perfect randomness.

By gradually increasing the number of compounds used for training, we were able to characterize the LCOCV accuracy distribution to evaluate how many compounds were needed to properly train the model (i.e., saturation) and if a model is overfitting. We define saturation in this context as a model’s ability to robustly predict held-out compounds and stabilize even upon addition of more compounds into the training data. In particular, we observed a stark difference between the multiclass logistic regression model using *GeneSet*_*y1-y5*_ and our CoHEC model using the same feature set, In particular, the multiclass representation had an initial LCOCV accuracy of 56.7% (± 2.93%) fitting the model with a single compound per MOA and does not ever saturate as each additional compound results in notable gains in performance with a maximum LCOCV accuracy of 99.1% using a maximum all 12 available compounds per MOA and all of the 713 pairwise DGE profiles (S5A Fig). In contrast, our CoHEC model using the same feature set, attained an initial LCOCV accuracy of 81.7% (± 2.72%) fitting the model with a single compound per MOA and surpasses the multiclass model’s performance using a maximum of only 7 compounds per MOA upon saturation with an average of 448/713 pairwise DGE profiles (Fig 1D). Put simply, given the same amount of information, CoHEC models can learn predictive patterns faster and more robustly than the direct multiclass adaptation. With this, the CoHEC model surpasses the multiclass adaptation performance using 37% less data. Although our *Clairvoyance*-optimized multiclass logistic regression models fit using *GeneSet*_*Multiclass*_ could predict MOA with high LCOCV accuracy (> 96%), we observed a lower benchmarking performance than our CoHEC with an initial LCOCV accuracy of 67.4% (± 2.78%) using a single compound per MOA and did not observe classification saturation until about 10 compounds per MOA. Our CoHEC model can outperform its multiclass counterparts and, therefore, derive more meaning given the same input data.

### Interpreting trained models

**Fig 2:**
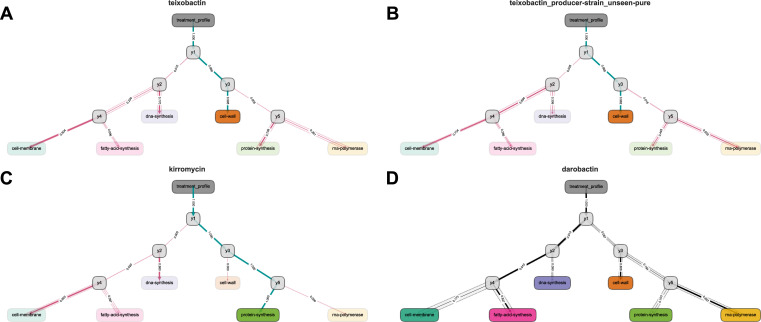
CoHEC model decision graphs for pure compounds, producer extracts, and darobactin representing MOA predictions. Prediction paths where each terminal colored node depicts a MOA, each internal gray node represents a sub-model decision point, and the edge-width corresponds to the probability according to the model for the respective path. Opaque halos around the edges represent SE with a large width corresponding to higher variance and vice versa. Rose and teal colored edges illustrate predictions traversing incorrect and correct paths, respectively, with black edges representing paths within a novel MOA paradigm. (A,B) Show teixobactin as a pure compound and the respective producer-strain while (C) depicts kirromycin and (D) represents darobactin. All of the prediction paths shown have no instance of the compound being previously observed by the model.

**Fig 3:**
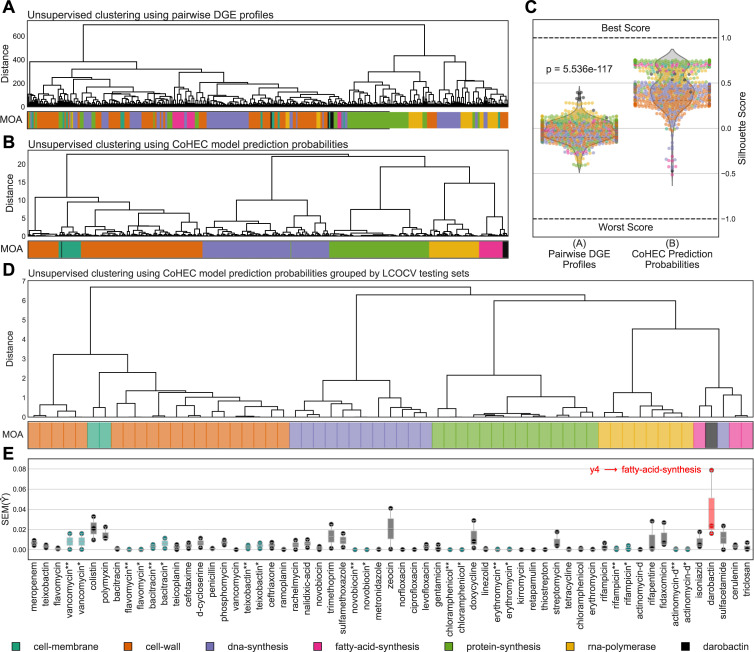
Unsupervised clustering performance and error profiles of transcriptomes and CoHEC model probability vectors. Unsupervised hierarchical clustering using (A) pairwise DGE profiles prior to feature selection (N = 3065 genes), (B) CoHEC model LCOCV test set prediction probabilities concatenated for all sub-models, and (D) CoHEC model prediction probabilities averaged by LCOCV test set. All hierarchical clustering uses Euclidean distance and ward linkage. C) Distributions of silhouette scores for (A) and (B) clustering results with Wilcoxon signed-rank test for statistical significance. D) Unsupervised hierarchical clustering and E) standard error profiles for each of the sub-model and the predicted path with red showing darobactin as a novel MOA and teal showing producer-extracts. Producer-strain extracts where the pure compound: (*) has been observed; and (**) has not been observed by the HEC model in the training data. The box plots extend from the Q1 to Q3 quartile values of the data, with a line at the median (Q2), and whiskers at 1.5 * IQR.

Interpretability of trained models is paramount in XAI and CoHEC models provides substantial insight into the decision-making process. For instance, fitted HEC models produce an array of probabilities for each of the 5 sub-models (*y1*-*y5*) with built-in methods designed to calculate the probability for traversing each of the 10 decision paths and to visualize the predictions via decision graphs (Fig 2 and S4 Table). In this case, the probabilities represent binary decision paths from each of the 5 logistic regression sub-models (though other algorithms for sub-models are supported) and the standard error is calculated for profiles grouped by LCOCV test set; that is, all associated pairwise DGE profiles corresponding to a compound in a LCOCV test set. These 10 probabilities computed by the CoHEC model on LCOCV test sets are machine informative as unsupervised analysis of these probabilities clusters compounds by MOA with statistically greater homogeneity than the input data of pairwise DGE profiles (Figs 3B, 3D and S1); further shown when comparing silhouette score distributions (Fig 3C). The ability of our CoHEC model to compute probabilities that can confidently cluster a compound with its respective producer-strain extract in a completely unsupervised setting provides a powerful avenue to dereplicate known compounds in high throughput (Fig 3D and 3E).

The CoHEC model was field-tested by examining crude extracts from producer-strains for 9 compounds as we would implement in a practical antimicrobial discovery pipeline. Even when the respective pure-compound had not been observed by the model in the training set during our LCOCV procedure, the classifier accurately predicts these producer-strain extracts and this holds true for texiobactin as well; a recently discovered inhibitor of cell-wall biosynthesis [8] (Figs 2A, 2B and S3, and S4 Table). We observed an agreement of probabilities and standard errors for the prediction paths between a pure compound and the associated producer-strain extract suggesting the model is resilient to potential noise from metabolites present in the extracts; further supporting dereplication applications. This ability to predict primary MOA at the level of crude fermentation extract relieves the bottleneck of purification and isolation of active compounds in natural product antibiotic discovery, addressing our objectives of providing a high throughput method for primary MOA determination and compound dereplication. Our CoHEC model was also able to accurately predict kirromycin, a known protein-synthesis inhibitor via EF-Tu [26], even though it was not used during our training process (Fig 2C). Finally, a compound with a newly discovered MOA not present in the training data, darobactin [27], was examined. Simulating a novel MOA is difficult because the paradigms are entirely dependent on the input data, each having unique properties, but our examination of standard error profiles reveal a method for identifying novel MOA (Fig 2D). While the CoHEC probabilities for darobactin point to a fatty-acid-synthesis inhibitor, the standard error profile along the predicted path is the highest observed in the entire dataset, particularly at sub-model *y4* in discriminating between fatty-acid-synthesis and cell-membrane inhibitors (Fig 3E). The routing, albeit error prone, towards fatty-acid-synthesis and cell-membrane inhibition is biologically relevant as darobactin uniquely targets the β-barrel assembly machinery, the BAM complex, which is necessary for outer membrane protein biogenesis [28]. This one-off novel target prevents proper modeling of robust cutoffs in standard error for rejecting a prediction. However, this instance proves that a negative result contains immense value and can be leveraged for identifying new chemical entities with novel activity. When the CoHEC model fails to confidently classify an antibacterial compound, assuming proper data preprocessing, it likely has a novel MOA or target. While the CoHEC model has high accuracy at predicting primary MOA for known compounds, it also proves robust when identifying compounds within a MOA and compounds representing new MOA such as kirromycin and darobactin, respectively.

Interpreting models based on gene expression data is difficult as this approach often captures downstream effects. Regardless, the decision graphs and sub-model gene coefficients are biologically relevant when evaluated via *Gene Set Enrichment Analysis* [GSEA] [29]. For instance, coefficient-ranked genes from sub-model *y2* (DNA-synthesis vs. *y4*) are enriched in both DNA and membrane-related GO terms (GO:0009432, GO:0006281, GO:0009102, GO:0006974, GO:0090305, GO:0009314, GO:0004518, GO:0006310, GO:0003677) while *y4* (cell-membrane vs. fatty-acid-synthesis) is enriched in membrane-related (GO:0006810, GO:0005886, GO:0016020, GO:0016021) and transport (GO:0006810) GO terms as shown in S5 Table. We also observed several nucleotide-binding related (GO:0003677, GO:0000166), transcription regulation (GO:0006355), protein-binding (GO:0005515, GO:0042802), and metal-binding (GO:0000287, GO:0046872, GO:0008270, GO:0051539) GO terms enriched in sub-model *y5* in the classification between protein-synthesis and rna-polymerase inhibitors.

### Extending methodology to datasets of other microbial strains and feature modalities

To evaluate our methods relative to other published antibiotic discovery work, we used a collection of transcriptomics and metabolomics datasets classifying MOA utilizing different microbial strains and feature modalities than used in this study. Hutter et al. 2004 generated a database of *Bacillus subtilis* transcriptional responses to treatments of 37 well-characterized antibacterial compounds from different MOA which were used to build a support vector machine model to predict MOA of antibacterial compounds. The training data from Hutter et al. 2004 [19] was not published in any public database. However, the support vector machine modeling approach and data transformations were well-documented so we used their methodology on our dataset to compare method performance. The methods from Hutter et al. 2004 applied to our dataset resulted in 54.5% (no normalization), 60.6% (TMM normalization), and 75.8% (log_2_ transformation) LCOCV accuracies (Tables 3 and S6), which is substantially lower than our CoHEC model. However, the Hutter et al. 2004 methodology used an unconventional approach that concatenates samples with respect to the feature axis, thus, increasing feature dimensionality and lowering the numbers of observations available for training. We used a more standard approach (i.e., stacking replicates on the observation axis instead of feature axis) in implementing support vector machines to evaluate the performance using modern methodology but this only increased the LCOCV accuracy by 3% (Table 2).

Next we tested our methods on external datasets by re-analyzing the transcriptomic data from Zoffmann et al. 2019 [12] and metabolomic data from Zampieri et al. 2018 [21]. Zoffmann et al. used a combination of transcriptomics and cell imaging data to predict 7 MOA in a different *E*. *coli* strain (see *Materials and Methods*). Zoffmann et al. did not publish the cell imaging data used to construct predictive models but we were able to download the counts from *NCBI Gene Expression Omnibus* (Accession: GSE110137) consisting of *E*. *coli* BW25113 challenged with 16 compounds. However, because of this inability to access the same data the Zoffmann et al. models cannot be directly compared to the results in our study. With the available public data, we computed pairwise DGE profiles, built CoHEC models, and optimized each sub-model using *Clairvoyance* with the same protocol and commands used to construct the CoHEC model in this study. The CoHEC model for the Zoffmann et al. 2019 transcriptomic data resulted in 100% LCOCV accuracy using only 35 gene features (S6 Table). However, Zoffmann et al. 2018 implemented a random forest classifier which we also implemented as shown in Table 2; though, CoHEC models out-performed this method and other standard classifiers.

The Zampieri et al. 2018 study had the most complete data that was publicly available, accessible through *EMBL-EBI BioStudies* (Accession: S-BSST113)[21]. This study used an iterative hypergeometric test to model metabolite responses of *Mycobacterium smegmatis* exposed to 62 compounds representing 18 MOAs. The Zampieri et al. metabolomic data had both a temporal aspect and contained a reference solvent for each timepoint. We constructed two CoHEC model paradigms: (*reference_t0*) treatment at ***t***_***n***_ vs. treatment at ***t***_***0***_; and (*reference_solvent*) treatment at ***t***_***n***_ vs. solvent at ***t***_***n***_ as both are biologically informative. We adapted our LCOCV strategy to incorporate treatment concentration for MOA that contained only a single representative. With this dataset, our CoHEC models achieved a LCOCV between 94.9% - 99.1% LCOCV accuracy with 492 metabolite features when using *t* = 0 as a reference and 88.2% - 96.3% with 494 metabolite features when using solvent as a reference (Tables 3, S6 and S7). Zampieri et al. 2018 [21] reports their performance using area under the curve, which is undefined for LCOCV, thus we were not able to directly compare model performance.

## Discussion

The CoHEC models present a purely data-driven XAI approach that can predict the primary MOA from unobserved compounds with high performance. This data-driven AI maximizes the available information content by asking simple questions about specific genes in a particular order to effectively evade statistical artifacts that are inherent in biological datasets where features greatly exceed the number of observations. We demonstrate the resourcefulness of our CoHEC methodology by comparing multiclass models either using the same input data (*GeneSet*_*y1-y5*_) or with gene sets designed for multiclass models (*GeneSet*_*Multiclass*_) and evaluating the number of compounds needed per MOA to stabilize prediction performance. The CoHEC model can exceed the performance of a multiclass model using the same base algorithm (e.g., logistic regression in this study) with only a fraction of the training data when using the same input features. This is desired in the field of bioinformatics where sample collection is a limiting factor and interpretability is key.

Furthermore, our hierarchical classification scheme is intuitive in that we can visualize the flux of weighted decisions through the graph for both individual and grouped observations (Figs 2 and S3 and S3 and S4 Tables). Most importantly, our approach does not sacrifice performance for interpretability because the CoHEC model can be unpacked to reveal feature weights that directly translate the AI decision process into human comprehensible terminology.

As membrane/transport GO terms were expected to be enriched in gene sets that classify MOA targets related to cellular structure and nucleotide/protein binding related terms were expected for gene product synthesis, we were not expecting a multitude of metal ion related GO terms in the classification of protein-synthesis and rna-polymerase inhibitors. However, this agrees with previous studies that have focused on metal-responsive ECF sigma factors, several of which are activated by iron depletion or by an excess of other metals such as zinc [30]; thus, overlapping with the GO terms enriched in our GSEA analysis (S5 Table). Bacterial ECF sigma factors are directly involved in the transcription process by recognizing promoter sequences, together with the core RNA polymerase enzyme, and initiate the transcription of the genes they regulate [31]. Although our models can be fully understood from a mathematical perspective, biological interpretation is limited to previous empirical studies and the extent of domain knowledge available. However, our methods are expected to provide a powerful resource in guiding empirical validation experiments to demystify complex biological processes.

While some multiclass classification problems do not require the architecture of hierarchical methods (e.g., S1A Fig), many more likely do given that negative data-mining results are rarely published. Our methods allow each decision to be evaluated and optimized with flexibility in classification algorithms, custom cross-validation-based objective functions, feature selection optimization, and hyperparameter tuning for each sub-model (S2 Table). In addition, the estimators of each sub-model could be further incorporated into ensemble methods such as tree-based gradient boosting for non-linear discrimination (e.g., XGBoost [32], CatBoost [33]) or AdaBoost [34] with logistic regression to further boost performance. Ultimately, the implementations developed here are widely adaptable to a variety of research goals where mining descriptive features for discriminating groups or complex classifications are desired. For instance, *Clairvoyance* was developed and validated on primary antibiotic MOA predictions but nascent versions of this algorithm were implemented to identify genes and pathways associated with cyanobacteria-moss symbiotic events [35], some of which have been experimentally validated *post hoc* using gene knockout experiments, demonstrating broad usage. We further demonstrate versatility by applying our methods to predict MOA from *E*. *coli BW25113* transcriptomics and *M*. *smegmatis* metabolomics timeseries (Tables 3,S6 and S7). In these demonstrations, we reveal that our AI determined a mix of logistic regression and non-linear tree-based classifiers to be optimal for predicting MOA. Furthermore, we expand the methods to investigate metabolomic profiles both in relation to solvent (*reference_solvent*) and to a baseline timepoint prior to antibiotic treatment (*reference_t*_*0*_) showcasing how one could investigate MOA from different biological contexts. Our methods outperformed these studies regardless of strain, species, modality, or large number of MOA categories while using a robust LCOCV accuracy metric.

Many empirical MOA classification experiments are based on macromolecular synthesis assays with limited targets and response variables. An open scientific question prior to this study was whether compounds with the same MOA determined by enzyme assays elicit the same whole transcriptome response. The lack of consensus statistically significant differentially expressed genes within a primary MOA (S1C Fig), the disparity of the global transcriptome in response to compounds both within and between MOA (Fig 3A), and the intermixing in multivariate analyses (S1B Fig) all suggest that concept of a MOA is a non-discrete fuzzy categorization. Although this presents challenges for classification algorithms, it also illuminates that there is unexplored functional space of new chemical entities within existing MOA that needs to be surveyed as compounds within a MOA can have very different biological effects. The 3 MOA with the lowest number of compounds in this study, cell-membrane (n = 2), fatty-acid-synthesis (n = 3), and RNA-polymerase (n = 4) are underrepresented in our training data because there is a very limited number of FDA-approved compounds (Tables 1 and S1) for these MOA. With the proper experimental design, our methodology could identify novel targets within these underrepresented MOA and expand the map of each MOA landscape and, in doing so, our understanding of antimicrobial resistance as a whole. In addition, a natural extension to our discovery methods would be to build secondary *post hoc* model as a successive layer to the main CoHEC model able to determine if and how a successfully classified compound is functionally divergent from previously observed compounds. A companion study examining sub-MOA diagnostic features may address the research required to execute such an addition [36]. In future work, we plan to empirically validate our model predictions and expand the underrepresented MOA classes to fortify the AI’s understanding of MOA-specific patterns.

As we have demonstrated, the data-driven methods used here are designed to be transferrable to other organisms as our primary goal was to rapidly screen a broad range of compounds with any antimicrobial activity. The specific methods developed here need no specialized equipment beyond access to a sequencing core, which is near universal, but we also demonstrate usage in other modalities such metabolomics. This is a benefit over previous methods that required extensive numbers of genetically modified reporter strains [11], mass spectrometers [16,21], or high-end microscopes [13]. Ultimately, this method is easily utilized by other researchers and the algorithms have been designed to flexibly accommodate model updates by automating feature selection, determining hierarchical structure, and parameter tuning with parallel-computing scalability for use on personal laptops to high-performance compute servers. Progressive science is built on open-sourcing knowledge, which is why we used an inexpensive publicly available strain, a detailed experimental design, and hosted the algorithms with tutorials demonstrating usage in an open-sourced programming language. These are components that facilitate an organically collaborative community resource accelerating antimicrobial discovery in both biology-centric and data-driven paradigms. Given our conclusion that there are likely unexplored spaces within existing MOA, such an effort should yield new chemical entities with novel activity and provide a unique perspective in data-mining for other researchers.

## Materials and methods

### Selecting antibiotic compounds

An initial set of antibiotics to be tested was chosen to represent the breadth of FDA-approved antibiotics across MOA classes and then certain MOA classes were supplemented with non-approved compounds with known antibiotic MOA to improve diversity. Subsequently, this compound set was dereplicated according to structural diversity using an ordination based on the molecular descriptors of the compounds [36].

### Crude extract production of antimicrobial-producing microbial strains

To further test the predictive model capabilities, strains producing known antibiotics were fermented and the whole broth was processed to produce crude extracts as described previously in a parallel study to this research [36] (S1 Table).

Strains were inoculated from a frozen glycerol stock onto SMSR4 agar plates (0.125 g casein, 0.1 g potato starch, 1 g casamino acids, 100ml R4 fermentation medium, 20 g bacto-agar in 1 L water). Morphology was confirmed under a 10X magnification using a Zeiss Stemi 2000 microscope and inoculated into 20ml of Modsb (15 g glucose, 10 g malt extract, 10 g soluble starch, 2.5 g yeast extract, 5 g casamino acids, and 0.2 g CaCl_2_-2H_2_O per 1 L deionized H_2_O, pH 7.0) in a 250ml flask, shaken at 150 rpm at 28^°^C for 2–5 days. Upon robust growth, the biomass was diluted 1:20 into 500ml of production medium R4 (10 g glucose, 1 g yeast extract, 0.1 g casamino acids, 3 g proline, 10 g MgCl_2_-6H_2_O, 4 g CaCl_2_-2H_2_O, 0.2 g K_2_SO_4_, 5.6 g TES free acid (2-[[1,3-dihydroxy-2-(hydroxymethyl)propan-2-yl]amino]ethanesulfonic acid) per 1 L deionized H_2_O, pH 7) for all strains except X4251. X4251 was diluted 1:20 into 500ml of production medium BPM (20 g glucose, 10 g organic soy flour (Bob’s Red Mill), 10 g pharmamedia (Traders Protein), 1 g (NH_4_)_2_SO_4_, 10 g CaCO_3_, 20 g glycerol per 1 L deionized H2O). Activity was monitored by bioassay and the active cultures were harvested between 4 and 7 days of growth in the production medium at 150 rpm at 28^°^ C. Crude extracts were generated by extracting the whole broth culture with an equal volume of water saturated n-butanol for 3 hours at room temperature and sonicated in a water bath for 20 mins prior to clarifying the butanol/aqueous layers with centrifugation. The n-butanol layers were removed into clean tubes and dried in a Savant Speedvac Concentrator heated to 45oC under vacuum. The dried substances were reconstituted and concentrated in 100% DMSO at 10X the original volume. Crude extracts were divided into 500ul aliquots, tested for MIC against W0153, and kept frozen until used for exposures to produce transcriptomes. The production of known compounds was confirmed with mass spectrometry, HPLC retention time, and/or spectrum of activity against resistant and sensitive test strains. Crude extracts were shipped on dry ice from NovoBiotic Pharmaceuticals to JCVI overnight.

### Antibiotic challenge experiments and sequencing

*Escherichia coli* strain W0153 (parent strain AB1157; asmB1 ∆tolC::kan modifications) was acquired from the Yale culture collection (http://cgsc2.biology.yale.edu/Strain.php?ID=4509). This modified AB1157 strain of *E. coli* has the *asmB1* allele, which reduces LPS synthesis, and the gene for *tolC* has been replaced by a Kanamycin resistance cassette. For the antibiotic challenge, 3 mls of *E*. *coli* strain W0153 at an OD_600_of 0.5, representing mid-log phase, were exposed to each antibiotic in biological triplicate at 1xMIC for 30 minutes. After 30 minutes of exposure, 100 μls of the cells were removed for OD_600_ values and CFU/ml counts (S4 Fig). This served as a checkpoint to observe that the 1xMIC antibiotic treated sample is showing an OD_600_ value and CFU/ml counts less than that than of the untreated control *t* = 30 minute solvent control but greater than that of the *t* = 0 sample, to ensure proper growth and to rule out an over treatment of the cells for an incorrect MIC. In parallel, the remainder of the cells were immediately pelleted at 4°C by centrifugation for 10 minutes at 2000 rpm in 1ml aliquots. The supernatant was removed and the pellets were immediately frozen in liquid nitrogen then stored for the RNA extraction processing at a later date. Total RNA was extracted by automation using the NucleoMag RNA extraction kit (*Macherey-Nagel*, *GmbH*) on the EpMotion Robotic liquid handler. For the resulting total RNA, RIN values were obtained to check for RNA quality using the 2200 TapeStation (*Agilent Genomics*, *Inc*.). Acceptable values to proceed to ribosomal subtraction were above a RIN of 5. Ribosomal RNA (rRNA) was subtracted from the total RNA to yield only messenger RNA for library construction using a bacterial rRNA depletion kit (*New England Biolabs*, *Inc)* at half reactions with a total RNA input maximum of 400 ng. The rRNA depleted product was quality controlled using an Agilent Bioanalyzer with the Agilent Pico chip for RNA detection to check for less than 0.5% of rRNA remaining. Then, 2.5 μl of the rRNA depleted samples, amounting to approximately 2–5 ng, is used as the input material to construct each cDNA library for RNA sequencing using the NEBNext Ultra Directional RNA Library prep kit (*Illumina*, *Inc*.) at half reactions. The resulting libraries were analyzed using Agilent High Sensitivity DNA chips to ensure library quality. Libraries were quantified and normalized by qPCR and then sequenced using the NextSeq 500 High Output Kit at 150 cycles producing approximately 9 million, 75 base-pair, paired-end reads for each library.

### Sequence processing, mapping, and normalization

Reads were quality trimmed and mapped to *E*. *coli* K-12 substr. MG1655 (Genbank: U00096.2, EcoCyc: v21.1) using *clc* (http://resources.qiagenbioinformatics.com) to produce a gene counts matrix (S8 Table). To maximize the number of observations and capture all of the variance in our dataset we used pairwise DGE profiles of the Trimmed Mean of M-values [TMM] normalized counts after filtering out a subset of genomic features. We removed low-quality samples that had fewer than 4000 detected genes or less than 100,000 reads mapping to non-ribosomal genes. The following genes were removed from the remaining samples: (1) genes other than rRNA whose abundance were sensitive to ribosomal depletion methods [G26 (D-galactose 1-dehydrogenase), G0-8867 (GcvB small regulatory RNA), EG30069 (RnpB RNA), G0-9281 (glutamate-pyruvate aminotransferase), and EG30100 (tmRNA)]; (2) rRNA genes; (3) non-protein-coding genes; (4) genes differentially expressed between comparisons of media and antibiotic carrier controls; and (5) genes differentially expressed in response to the producer-strain metabolic background (i.e. pure compound vs. producer extract). Our method of pairwise DGE is calculated by the following: (1) remove genes described above; (2) TMM normalization using *edgeR* [37]; and (3) for each compound in a sequencing run we calculate the log_2_(compound_r_)—log_2_(control_r’_) for all compound replicates ***r*** and respective control replicates ***r’*** using a pseudocount of 1 (S9 Table). Our dataset consists of 9 sequencing runs, each with several antibiotics representing different MOA, and we only include relationships within a sequencing run to minimize batch effects and reduce variance introduced from non-biological processes. Statistically significant differentially expressed genes were computed using *edgeR*’s exactTest with |log_2_FC| ≥ 2 and FDR < 0.001 to minimize the influence of off-target effects (S1C Fig).

### Hierarchical ensemble of classifiers modeling

The graphical structure of our HEC model is entirely data-driven to exploit natural patterns within the data. However, it is possible to use a predefined structure but, due to the limitations in our understanding of latent variables in biological classification tasks, we implemented an unsupervised method to allow the data to dictate the hierarchy. Our methods for implementing this unsupervised hierarchy alludes to the concept of an *eigengene* which, essentially, is the first principal component of a dataset using a subset of features [38]. In this context, we transpose the operation by generating *m*-dimensional *eigenprofiles* representing each MOA class from our pairwise DGE feature matrix [***X***] (m = the number of genes). We then use classical agglomerative methods with Euclidean distance and ward linkage to cluster these profiles revealing the relationships between MOAs as a natural hierarchical structure entirely dependent on the differential expression profiles. The implementation for this pipeline can be found within the soothsayer.hierarchical.Topology object.

Once the structure is determined, the framework resembling a decision-tree is used to construct a directed *NetworkX* [39] directed graph where each internal or terminal node in the graph represents a sub-model or classification category (e.g. MOA), respectively. The *paths* for each classification target in the directed graph and the target matrix [***Y***] can be obtained from the soothsayer.hierarchy.Topology object using the get_paths and get_target_matrix methods, respectively. The resulting *paths* contain a collection of ordered nodes when traversing the graph towards the desired target classification from the input node. ***Y*** contains the binary classifications for each sub-model in the graph and is used with ***X*** to train all of the sub-models simultaneously using the fit method. The model object is implemented in soothsayer.classification.HierarchicalClassifier and mimics the API of *scikit-learn* (arXiv:1309.0238).

Each sub-model node serves as a vessel for storing custom fields including feature sets (e.g., gene subsets from feature selection), feature matrices (e.g., gene expression or pairwise DGE data), and *scikit-learn* compatible classification models among other custom data fields. The edge weights between nodes in the graph contain probabilities from the parent sub-models and these can be examined quantitatively or visualized for a qualitative assessment of a single prediction or group of predictions (e.g., replicates) with standard error of the mean error bar support. The sub-model nodes contain a unique classification model equipped with custom model hyperparameters and gene subsets designed to optimize a specific classification task in the overarching model. The sub-model hyperparameters and gene sets are shown in S2 Table.

This architecture allows maximum flexibility for decomposing complex predictions into a sequence of simple predictions with any set of features or any *scikit-learn* compatible classification model. The implementations for preprocessing data, determining hierarchical structures, building and evaluating hierarchical classification models, and analyzing diverse datasets can be found within our *Soothsayer* Python package. Additional supervised-classifier algorithms, as shown in Table 2, were implemented and evaluated using *scikit-learn* with random_state = 0 when applicable.

An example of the CoHEC prediction process for the transcriptomic response to teixobactin (Fig 1C): (1) evaluate using the 102 genes in sub-model *y1* with 99% probability diffusing towards sub-model *y3*; and (2) sub-model *y3* uses a subset of 101 genes, of which only 10 genes overlap with sub-model *y1*, routing the transcriptome profile to the cell-wall MOA with 99.5% probability as a terminal classification.

### Simulating novel compounds by evaluating model on unobserved compounds via Leave Compound Out Cross Validation

In the context of drug discovery, our LCOCV training and testing splits simulated the following scenarios: (1) if there is only a pure-compound then we leave out all profiles for the compound in the test set to simulate an unobserved compound; (2) if there are both producer-extracts and pure compounds we (2a) leave out all profiles related to the compound and test only on the pure compound (again, simulating an unobserved compound); (2b) leave out all profiles related to a compound and test only the producer-extract (simulating an unobserved compound derived from extract); and (2c) leave out only the producer-extract from the test set (simulating a known compound derived from extract). With this scheme, we end up with 59 unique LCOCV training and testing splits (Fig 3D and 3E and S4 Table).

### Feature selection (Clairvoyance)

*Clairvoyance* is a novel feature selection algorithm designed to enrich a dataset for features that maximize an accuracy-based objective function. In the case of this study, the feature selection is applied to pairwise DGE profiles to identify gene sets that optimize classification accuracy for the specific binary classification task associated with each sub-model. The methods in *Clairvoyance* extend on concepts inspired by Zakharov and Dupont 2011 [40] and Warshan et al. 2017 [35] by adding pseudo-random sampling preserving class proportions, iterative processes, subsetting feature weights from classifiers with an accuracy threshold, and the use of both decision tree- and logistic regression-based ensembles for versatile performance. *Clairvoyance* implements parallel computations that are scalable and can be configured for running quickly on local machines for notable performance gains or exhaustively on compute clusters for even greater boosts in performance. The *Clairvoyance* algorithm is available in our *Soothsayer* Python package implemented as 1) a low-level object for prototyping in interactive consoles as soothsayer.feature_extraction.Clairvoyant and (2) a stand-alone executable with very few dependencies.

The objective function of *Clairvoyance* maximizes the cross-validation accuracy which can accept custom cross-validation training/testing pairs; in this case, leaving all instances of a compound out for a testing set (i.e., LCOCV). In the context of drug discovery, our objective function was to maximize LCOCV accuracy of held-out compounds to simulate the performance on novel compounds unobserved by the model.

The basic strategy of *Clairvoyance* is as follows: (1) iterate through a grid of hyperparameter configurations and for each iteration ***k*** construct classifier ***clf***_***k***_; (2) shuffle the training data without replacement into equally sized observation subsets ***A*** and ***B*** while maintaining class proportions to produce (***X***_***A***_**, *y***_***A***_) and (***X***_***B***_**, *y***_***B***_) training/testing pairs, respectively; (3) train ***clf***_***k***_ on (***X***_***A***_**, *y***_***A***_) and predict on (***X***_***B***_**, *y***_***B***_); (4) train ***clf***_***k***_ on (***X***_***B***_**, *y***_***B***_) and predict on (***X***_***A***_**, *y***_***A***_); (5) store the weights (i.e. *|coefficients****|*** for logistic regression or *feature importances* for tree-based models) of each feature (e.g. gene), the accuracy of the held out subset, and the hyperparameters of ***clf***_***k***_ for the fitted models from steps (3 and 4); and repeat steps (1–5) ***n_iter*** times for each hyperparameter configuration ***k***. More specific algorithm details can be found in the *S1 Methods* and in the open-sourced code.

The hyperparameter grid for logistic regression includes the following: (C) inverse of regularization strength; and (penalty) the penalization type as either *L1* or *L2* regularization. For decision tree classifiers, we include the following hyperparameters: (criterion) the function to measure the quality of a split with *gini* for Gini impurity or *entropy* for the information gain; (max_features) the number of features to consider when looking for the best split; and (min_samples_leaf) the minimum number of samples required to be at a leaf node.

The weights for each fit are collected in an array and reduced into a single weight vector with features listed in descending ordered by their predictive capacity. Each feature is iteratively added and the classifier is cross-validated using either custom training/testing sets or randomly generated stratified *K*-fold splits serving as the objective function maximized by *Clairvoyance*. An ***early_stopping*** parameter is used to stop the algorithm from adding features if there have not been advancements in the accuracy for a user-specified number of iterations (100 in this study) to increase computational efficiency. Summary statistics are generated for the cross-validation performance of each feature subset and plots are generated showing the transitions between feature subsets with respect to cross-validation classification accuracy.

The basic form of the algorithm can be augmented by running the cross-validation methods on models with accuracy levels above a particular threshold, using either logistic regression and/or tree-based methods, and iteratively feeding enriched subsets into the algorithm for exhaustive data-mining to maximize the performance of the feature selection. These methods can be configured for single run use or in a pipeline that runs all configurations and produces a synopsis of all executions sorted by the highest performing run. A major component of the algorithm’s flexibility is the incorporation of both logistic regression and decision trees for the objective function maximization as some discrimination tasks are better described by linear relationships of log-odds while other by non-linear criteria. The resulting feature subsets can be further explored using ensemble methods such as random forests and boosting ensembles with Bayesian or randomized hyperparameter tuning. We used the gene sets derived from *Clairvoyance* to build individual sub-models in our HEC model.

*Clairvoyance* identified several combinations of gene sets and hyperparameters of equally high accuracy using the 41-compound set listed in S1 Table (not including kirromycin or darobactin). To determine which gene sets and hyperparameter configurations would be used for each sub-model, we sorted each configuration by the following criteria and in this order: (1) ↓ LCOCV accuracy for 41 training compounds, (2) ↑ μ(standard error) of predicted path for 41 training compounds, and (3) ↑ number of genes; ↑ (lower is better) and ↓ (higher is better) refer to ascending and descending order, respectively.

### Evaluation methodologies and benchmarking

The hierarchical nature of our dataset allowed us to evaluate our methodology in multiple ways. Our data is arranged in the following hierarchy: pairwise DGE profile → transcriptome → compound → MOA as shown in Table 1. With this hierarchy, we are able to use LCOCV training and testing splits to evaluate our data. As we perform LCOCV, we stack the predictions for all of the test sets that were held out into an array and once we complete cross-validation we can compute the overall LCOCV accuracy we refer to as “Individual Pairwise Profiles Accuracy”. When we compute the accuracy of each LCOCV test set, we can also compute average accuracy of all the sets which we refer to as “LCOCV Test Set Accuracy”.

Our experimental design includes several replicates for each compound treatment. These replicates in combination with the pairwise DGE profiles result in several observations to predict for a given compound and can be grouped via majority-voting methods using either soft voting (averaged probabilities) or hard voting (only considering terminal predictions). In the case of this study, hard voting would translate to predicting each profile separately and then using the most common prediction while soft voting sums the sub-model probabilities for all of the replicates and averaging. Soft majority voting can be calculated by averaging the probability matrix **Ŷ** (Y_hat where rows are testing pairwise DGE profiles and columns are sub-model probabilities), ensuring the sub-model probabilities sum to 1.0, and traversing the path of highest probability. In Python, this operation is achieved by Y_hat.mean(axis = 0*)* where Y_hat is the output of the predict_proba method built into the HierarchicalClassifier object. This aggregated probability profile is used as input for the predict_from_probas method which yields the most probable path from a directed walk across the aggregated probabilities. Hard majority voting can be computed simply by computing the prediction with the most occurrences via y_hat.value_counts().idxmax() where y_hat is a collection of terminal predictions. If the most common predicted MOA is not unique then the prediction is rendered inconclusive via hard voting. Both majority voting methods have proven to be equally robust for predicting left-out compound. Our dataset allows us to use these majority voting approaches since we have discrete groupings (compounds) in each of our broader classification categories (MOAs). This grouping method only works for categories with discrete subcategories and, thus, does not apply to all classification tasks.

To provide deeper insight into model fitting characteristics, we benchmarked our models using two separate approaches. The first of which was by fitting the model with a variable number of random compounds from each MOA and evaluating model performance on the entire LCOCV set (Figs 1D, S5A, S5C and S5E). The second approach was by shuffling the MOA labels and fitting these null models on the shuffled dataset (Figs 1E, S5B, S5D and S5F). For both approaches, we repeated this process for 500 iterations to obtain a distribution of values instead of single point estimates. We used the same 500 random seeds in our sampling for each of our 3 MOA models, which use the same pairwise DGE profiles albeit different gene sets as either *GeneSet*_*y1-y5*_ or *GeneSet*_*Multiclass*_, making our benchmarking results comparable between methodologies.

### Gene set enrichment analysis

Sub-model specific gene sets were evaluated using the logistic regression coefficients from each fitted sub-model against the *Gene Ontology* database (http://geneontology.org/docs/download-ontology/ | go.obo [format 1.4, releases/2019-05-09]) using *GSEA’s Prerank* module with 1000 permutations [29]. The gene sets were extracted from *EcoCyc* v21.1 flat files (data/gene_association.ecocyc). Significance is determined by FDR < 0.25 as suggested by the *GSEA* documentation.

### External studies and datasets

We evaluated our methods using 3 external studies including Hutter et al. 2004 methods (no data available), Zoffmann et al. 2019 (transcriptomics), and Zampieri et al. 2019 (metabolomics). Each dataset contained their own caveats for analysis. Hutter et al. 2004 did not publish data but described modeling methodology. In this case, we were able to reproduce model methodology but not use the same data therefore we could not directly compare results. The Zoffmann et al. 2019 study did not publish cell imaging data used for modeling but did publish an auxiliary transcriptomics dataset that we were able to leverage for modeling. However, the Zoffmann et al. 2019 transcriptomics dataset had several MOA with only a single compound making the use of robust evaluation such as LCOCV impossible. To adapt their dataset to our stringency, we used slightly broader MOA categories by adjusting as follows: {“cell wall synthesis inhibitor / lipoprotein”, “cell wall synthesis inhibitor”} → “cell-wall” and {"DNA replication inhibitor", "DNA damage", "Folic Acid synthesis inhibitor"} → “dna-synthesis” so we could have more than one representative per MOA. Zampieri et al. 2018 was the most comprehensive and accessible dataset. We were able to obtain metabolomic profiles but since we used LCOCV accuracy, we could not directly compare to their AUC scores as AUC is undefined for LCOCV. To properly integrate the zscore-normalized metabolic data with our methods, we used pairwise differences instead of pairwise DGE profiles as these were the most comparable.

## Supporting information

S1 Fig Unsupervised clustering and marker gene signatures. Model dataset (TCGA-PANCANCER, http://archive.ics.uci.edu/ml/datasets/gene+expression+cancer+RNA-Seq) and (B) our training data from this study. A) Shows the discrimination of 5 different cancer types based on gene expression patterns for 801 samples with 20531 gene features. In this scenario, “out-of-the box” standard multivariate statistical analyses are sufficient to differentiate the cells types with high confidence. (B) Shows the results for the same multivariate analysis using the training data from this study which includes the transcriptional response to 41 compounds in 6 categories from 713 observations and 3065 gene features represented using Principle Component Analysis ordination. Multivariate statistical analyses cannot discriminate these samples by their MOA. (C) Differentially expressed genes [DEGs] shared by different compounds within a MOA. The low proportions (y-axis) shows that different compounds within a MOA have a different transcriptional response in terms of DEGs and that there is not a clear diagnostic profile for each MOA.
(EPS)

S2 Fig XAI pipeline for determining hierarchical structure, selecting gene feature sets, and building MOA classifier. The pipeline begins with a basic next generation sequencing procedure for generating the training data for our method which includes treatment of organism with a compound of interest, transcriptome sequencing, read mapping to reference, filtering data, and generating pairwise DGE profiles (refer to *Materials and Methods*). The remainder of the pipeline is domain agnostic and is broadly applicable. The training data consists of a feature matrix **X** (e.g. the pairwise DGE profiles) and the target vector **y** (e.g. MOA classification). The training data is fed into *Soothsayer*’s *Topology* object which determines the hierarchical structure of the HEC model. Each of the sub-models at internal nodes within the tree-like structure undergo a feature selection procedure via *Soothsayer*’s *Clairvoyance* algorithm. This procedure determines a gene subset that optimizes the accuracy for the performance of the sub-model. Next, the: (1) sub-model estimators; (2) sub-model specific gene feature sets; and hierarchical structure are fed into *Soothsayer’s Hierarchical Classifier* object to build the CoHEC model. This CoHEC model is a MOA classifier and can be validated by removing all instances of a compound from the training data, training the model on this subset, and then testing the model’s MOA prediction accuracy with the left-out profile subset.
(EPS)

S3 Fig MOA- and compound-specific model LCOCV prediction accuracy. MOA-specific prediction accuracy for unobserved compounds with a heatmap (left) showing the accuracy for each compound at each sub-model in the CoHEC model. Bar chart (right) showing the mean accuracy for each compound for the terminal prediction of the CoHEC model colored by MOA. Producer-strain extracts where the pure compound: (*) has been observed; and (**) has not been observed by the CoHEC model in the training data. Error bars reflect standard error of mean unless specifically noted otherwise.
(EPS)

S4 Fig Survival rate for compounds. Percent survival for various compounds compared to solvent controls measured at *t* = 30 minutes via optical density of sample at wavelength of 600 nm. Error bars are standard deviations taken from 3 biological replicates with 3 technical replicates each. Crude extract indicated by [CE] suffix.
(EPS)

S5 Fig Model performance and benchmarking for multiclass MOA and 30S/50S protein-synthesis classifiers. Benchmarking of *Clairvoyance*-optimized (A) multiclass logistic regression MOA and (B) binary 30S/50S protein-synthesis sub-MOA model performance (N = 500 permutations without repetition) showing (upper) the number of compounds included during (lower) LCOCV evaluation relative to performance. Error bars represent standard error of mean. Kernel density of LCOCV accuracy for (B) multiclass MOA and (D) binary 30S/50S null models (N = 500 permutations without repetition) and dashed horizontal lines representing actual model performance.
(EPS)

S6 Fig Gene set overlaps between MOA predictive model feature selection. Upset plots showcasing gene set overlap of CoHEC sub-models, multiclass MOA, and binary 30S/50S feature selection.
(EPS)

S1 Table Antibiotic compound list with MICs and MOA categories. Each of the 41 antibiotics used in the training data with minimum inhibitory concentrations and MOA categorization.
(XLSX)

S2 Table Sub-model parameters, features, and performance. Classifier parameters for *scikit-learn* estimators for each sub-model along with gene sets and performance metrics before and after optimization.
(XLSX)

S3 Table Sub-model-specific gene sets with functional annotations and weights. Gene sets used CoHEC model derived from *Clairvoyance* feature selection with fitted logistic regression coefficients and *EcoCyc* annotations. Additionally includes multiclass MOA model and 30S/50S protein-synthesis inhibitor model coefficients.
(XLSX)

S4 Table CoHEC model prediction probabilities for cross-validation and test sets. Prediction probability paths from cross-validation and test set combinations with respect to each sub-model. Cross-validation training and testing pairs for each sub-model included.
(XLSX)

S5 Table Gene set enrichment analysis of fitted sub-model coefficients. Results from *GSEA’s Prerank* module using coefficients as ranked weights with gene sets from the Gene Ontology database.
(XLSX)

S6 Table Sub-model parameters, features, and performance for external datasets. Classifier parameters for *scikit-learn* estimators for each sub-model along with gene sets and performance metrics before and after optimization. These parameters pertain to Zoffmann et al. 2019 and Zampieri et al. 2018.
(XLSX)

S7 Table Classification metrics. Classification metrics such as accuracy, f1 score, precision, and recall for different models and evaluation methods.
(XLSX)

S8 Table Gene expression counts. Unnormalized gene expression counts.
(TSV)

S9 Table Model training and testing dataset. Pairwise log_2_FC differential gene expression profiles.
(TSV)

S1 Methods *Clairvoyance* algorithm. Detailed description of *Clairvoyance* algorithm including parameters and operations.
(DOCX)
