# Supplementary material for: Predicting antimicrobial mechanism-of-action from transcriptomes: A generalizable explainable artificial intelligence approach
Source: PLoS Comput Biol. 2021 Mar 29;17(3):e1008857. doi: 10.1371/journal.pcbi.1008857 (PMC8031737; doi:10.1371/journal.pcbi.1008857)
Supplement: S1 Methods — Detailed description of Clairvoyance algorithm including parameters and operations. (DOCX) [file pcbi.1008857.s016.docx]

***Algorithm*:**

***Clairvoyance*** *–* Feature selection to identify features and model hyperparameters that optimize classification performance

***Inputs:***

***-X*** *(filepath) =* Feature table (n = observations, m = features)

-***y*** *(filepath) =* Classes (n = observations)

--***cv*** *(integer, filepath)* = The number of stratified *K*-fold cross-validation pairs or custom cross-validation pairs

***Hyperparameters:***

***Modeling:***

***--model_type*** (*string*) = Type of classifier used {logistic regression, decision tree classifier}

***--n_iter*** (*integer*) = Number of iterations to split data and fit models

***--min_threshold*** (*string*, *float*) = (Iterative mode uses a comma-separated list of values) Accuracy threshold for selecting features to use for agglomerative feature selection and cross-validation

***--percentiles*** *(string, float) =* (Iterative mode uses a comma-separated list of values) The upper n^th^ percentile of ranked feature weights to include in next iteration;

***--early_stopping*** *(integer)* = Stopping the algorithm if certain number of iterations do not increase the accuracy;

***Utility***:

***--n_jobs*** *(integer)* = Number of threads to use for parallelization;

***--random_state*** *(integer)* = Random seed used for reproducibility;

***--random_mode*** *(integer) =* Type of randomness to choose including using same random seed for each iteration, a different random seed for each iteration but repeated, or a different random seed for each instance;

***Outputs***:

- Summary tables including classifier type, hyperparameters, and (weighted) features;
- Configuration(s) with highest ***cv*** accuracy;

***Procedure (Iterative mode):***

Load ***X, y,*** and ***cv*** input data;

***X_initial_*** = ***X***;

Initialize ***kernel;*** an empty *N*-Dimensional labeled array to store, index, and summarize all analysis;

For (***model_type*** in ***model_types***):

For (***current_percentile*** in ***percentiles***):

If (***current_percentile*** is ***percentiles[0]*** (i.e. beginning of ***percentiles*** list))**:**

***X_percentile_*** = ***X_initial_***;

Else:

***X_percentile_*** = Feature table with n^th^ percentile of ranked features;

Construct ***model*** using ***Clairvoyant(model type, n_iter, random_mode, random_state);***

Fit ***model*** using ***model.fit(X_percentile_, y)*** *[Details below];*

For (***threshold*** in ***min_threshold)***:

***feature_weights*** = Sort ranked feature weights for models with accuracy > ***threshold*** in descending order*;*

***features*** = Feature labels from sorted ***feature_weights***;

***clf*** *= <****model_type****>* classifier using hyperparameters with highest accuracy

Initialize ***query_features****,* an empty list of features that is updated dynamically;

Initialize ***scores***, an empty list to populate with cross-validation scores;

For (***j*** in number of ***features***):

Append ***feature_j_*** to ***query_features;***

Compute ***score_j_*** from cross-validation results using ***clf, X_percentile_[query_features], y***, and ***cv***;

Append ***score_j_*** to ***scores;***

If (***score_j_*** is not higher than previous ***early_stopping*** scores)***;***

Stop agglomerative cross-validation;

Plot score curve with number of features on *x*-axis and scores on *y*-axis;

Store feature selection results in ***kernel*** indexed by ***model_type, clf, current_percentile***, and ***threshold****;*

If (***current_percentile*** is not ***percentiles[-1]*** (i.e. end of ***percentiles*** list))**:**

Write feature table for ***X_percentile_*** with n^th^ percentile of ranked features for next percentile;

***Procedure (Clairvoyant):***

Function ***f_weight_(****clf****):***

Assert ***clf*** model has been fitted;

If (***model_type*** is ***LogisticRegression***):

Return (Absolute value of coefficients)

If (***model_type*** is ***DecisionTreeClassifier***):

Return (Feature importances)

Initialize ***hyperparameter_grid;*** a dictionary of hyperparameters and values;

Initialize ***weights;*** an empty array to store weights;

Initialize ***scores;*** an empty array to store scores;

For (***iteration*** in ***n_iter***):

Set random ***seed*** as ***random_state + iteration*** for reproducible stochastic behavior;

Split ***X_percentile_*** and ***y*** into equally sized subsets ***A*** and ***B*** while considering class proportions in ***y****;*

For (***query_hyperparameters*** in ***hyperparameter_grid***);

Initialize ***clf*** as *<****model_type****>* classifier with ***query_hyperparameters****;*

*# Training=A, Testing=B*

***ŷ_B_*** = Predict with ***X_B_*** using ***clf*** fit with (***X_A_***, ***y_A_***);

***weights_B_*** = ***f_weight_***(***clf***);

Append ***weights_B_*** to ***weights***;

***score_B_*** = Calculate accuracy score with (***y_B,_ ŷ_B_***);

Append ***score_B_*** to ***scores***;

*# Training=B, Testing=A*

***ŷ_A_*** = Predict with ***X_A_*** using ***clf*** fit with (***X_B_***, ***y_B_***);

***weights_A_*** = ***f_weight_***(***clf***);

Append ***weights_A_*** to ***weights***;

***score_A_*** = Calculate accuracy score with (***y_A,_ ŷ_A_***);

Append ***score_A_*** to ***scores***;

Return (***scores*** and ***weights*** with respect to ***hyperparameter_grid***)
